# A comprehensive evaluation of human papillomavirus positive status and p16^INK4a^ overexpression as a prognostic biomarker in head and neck squamous cell carcinoma

**DOI:** 10.3892/ijo.2014.2440

**Published:** 2014-05-12

**Authors:** ZEYI DENG, MASAHIRO HASEGAWA, KAZUO AOKI, SEN MATAYOSHI, ASANORI KIYUNA, YUKASHI YAMASHITA, TAKAYUKI UEHARA, SHINYA AGENA, HIROYUKI MAEDA, MINQIANG XIE, MIKIO SUZUKI

**Affiliations:** 1 Departments of Otorhinolaryngology, Head and Neck Surgery, Graduate School of Medicine, University of the Ryukyus, Okinawa, Japan; 2 Public Health and Hygiene, Graduate School of Medicine, University of the Ryukyus, Okinawa, Japan; 3 Department of Otorhinolaryngology, Head and Neck Surgery, Zhujiang Hospital, Southern Medical University, Guangzhou, P.R. China

**Keywords:** head and neck cancer, human papillomavirus, HPV *E6*, *E7* mRNA, p16^INK4a^, prognosis

## Abstract

Head and neck squamous cell carcinoma (HNSCC) patients with human papillomavirus (HPV) infection have better prognosis than those without HPV infection. Although p16^INK4a^ expression is used as a surrogate marker for HPV infection, there is controversy as to whether p16^INK4a^ reliably indicates HPV infection. Here, to evaluate the accuracy of p16^INK4a^ expression for determining HPV infection and the prognostic value of HPV infection and p16^INK4a^ expression for HNSCC survival, especially oropharyngeal squamous cell carcinoma (OPSCC) survival, 150 fresh-frozen HNSCC samples were analyzed for HPV DNA, *E6/E7* mRNA and p16^INK4a^ expression by polymerase chain reaction and immunohistochemistry. p16^INK4a^ expression was scored from 0 to 4 according to the percentage of p16^INK4a^-positive cells, with overexpression defined as >40% positive cells. Of the 150 tumor samples tested, 10 tumors were nasopharyngeal, 53 oropharyngeal, 39 hypopharyngeal, 24 laryngeal and 24 were located in the oral cavity. HPV DNA was detected in 47 (31.3%) samples, but only 21 also exhibited HPV mRNA expression. Inter-rater agreement was low between p16^INK4a^ expression and HPV DNA presence and between p16^INK4a^ expression and HPV mRNA expression, but was good between the combination of HPV DNA status and p16^INK4a^ overexpression and HPV mRNA expression. Three-year recurrence-free survival was significantly higher for OPSCC patients who were HPV DNA-positive than for OPSCC patients who were HPV DNA-negative (P=0.008) and for OPSCC patients over-expressing p16^INK4a^ than for without overexpressing p16^INK4a^ (P=0.034). Multivariate analysis revealed that T1-3 stage and the combination of HPV DNA positivity and p16^INK4a^ overexpression predicted significantly better recurrence-free survival. This combination is a more accurate marker for active HPV infection in HNSCC than HPV DNA status or general p16^INK4a^-positive status alone and offers a useful and reliable method for detecting and determining the prognosis of HPV-related HNSCC.

## Introduction

Each year, 600,000 new cases of head and neck squamous cell carcinoma (HNSCC) are diagnosed worldwide (1). Common risk factors for most forms of HNSCC include heavy consumption of tobacco and/or alcohol (2), although the oropharyngeal squamous cell carcinoma (OPSCC) is less likely to be associated with tobacco and alcohol exposure and more often correlated with human papillomavirus (HPV) infection (3). The incidence of OPSCC associated with HPV infection is increasing; for example, among cases of tonsillar cancer in Stockholm, HPV-positive cases rose from 23% in the 1970s to 57% in the 1990s and 79% from 2000 to 2007 (4). Moreover, alongside tobacco and alcohol, high-risk HPV variants (HR-HPVs) have emerged as risk factor for HNSCC, including OPSCC (5).

HNSCC patients who are HPV positive have substantially better prognosis than those who are HPV negative (6–10). Although the detection of *E6/E7* mRNA transcripts is regarded as the gold standard for the presence of clinically relevant (active) HPV (11), the requirement of unfixed (fresh frozen) tissue and the cost of polymerase chain reaction (PCR) make direct detection of *E6/E7* impractical for cancer diagnostics at present. Accordingly, many studies have attempted to identify an easily measured surrogate maker for the diagnosis of HPV-associated HNSCC.

Expression of the tumor suppressor p16^INK4a^ has been proposed as a surrogate marker for HPV infection: its over-expression is thought to reflect the presence of biologically active HPV infection given that functional inactivation of pRb by viral E7 induces p16^INK4a^ upregulation. Detection of p16^INK4a^ expression can also be performed using formalin-fixed, paraffin-embedded (FFPE) samples (11–13). However, there is controversy as to whether p16^INK4a^ expression reliably indicates HPV infection (11,12).

Klaes *et al* classified p16^INK4a^ staining as negative (<1% of cells positive), sporadic (<5% of cells positive), focal (<25% of cells positive) or diffuse (>25% of cells positive) (14). Other studies have defined p16^INK4a^ expression in tumors as strong and diffuse when ≥70% of cells (cytoplasm and nuclei) are stained (15–17), while Fischer *et al* assessed tumors as p16^INK4a^ positive when ≥5% of cells were immunopositive (18). These diverse scoring systems may lead to significant discrepancies across studies in the relationship between HPV infection and p16^INK4a^ expression. Furthermore, p16^INK4a^ expression has been observed in tumor-free tonsillar tissue without HPV infection, implicating other mechanisms in p16^INK4a^ upregulation (19). Bussu *et al* concluded that it is unnecessary to measure a surrogate, such as p16^INK4a^ expression, for objective, reliable, and direct diagnosis because HPV nucleic acids can be detected by PCR without requiring subjective assessments by histopathologists (17).

In this study, we evaluated the relationship between HPV infection and p16^INK4a^ expression and the value of both HPV-positive status and p16^INK4a^ expression levels for HNSCC prognosis using tissue samples from a well-characterized cohort of Japanese patients with HNSCC receiving curative treatment. We measured the presence of HPV DNA, HPV *E6/E7* mRNA expression using fresh-frozen samples and measured p16^INK4a^ expression using FFPE samples.

## Materials and methods

### Subjects and study design

The eligibility criteria for this study were as follows: the presence of previously untreated, pathologically confirmed primary HNSCC without distant metastasis (M0); receiving curative treatment of surgery alone, surgery combined with radiation therapy (RT) or chemoradiotherapy (CRT), concurrent chemoradiotherapy (CCRT) or RT alone with >66 Gy of total dosage; and complete remission after primary treatment. The treatment modalities were determined according to tumor location, tumor stage, response to induction chemotherapy, and general physical condition, not by the results of HPV status and p16^INK4a^ in tumor tissue. Based on these criteria, 150 patients treated by the Department of Otorhinolaryngology, Head and Neck Surgery, University of the Ryukyus, Japan were recruited between October 2006 and June 2013. In contrast to our previous study on HPV status and squamous cell carcinoma antigen (SCCA), this study involved a greater total number of cases who met the inclusion criteria and we also updated the prognostic information of some of the patients reported previously (8). Each patient gave written informed consent before enrolment. The research protocol was approved by the Ethics Committee of the University of the Ryukyus. Tissue samples were snap-frozen in liquid nitrogen during biopsy or surgical excision and stored in liquid nitrogen until further analysis. Demographic and clinicopathologic parameters for each patient were collected at scheduled intervals during the follow-up period.

### Cell lines and culture

The cervical cancer cell lines CaSki (harboring ∼600 copies of integrated HPV-16 DNA/genome) and SiHa (1–2 copies of integrated HPV-16 DNA/genome) were purchased from the European Collection of Animal Cell Cultures (Salisbury, UK) and the American Type Culture Collection (Tokyo, Japan), respectively, and cultured according to the suppliers’ instructions.

### DNA and RNA extraction

Genomic DNA and total RNA were extracted from frozen tumor samples, SiHa cells and CaSki cells using the Gentra Purification Tissue kit (Qiagen, Germantown, MD) and the ToTALLY RNA^™^ kit (Ambion, Austin, TX), respectively, according to the manufacturers’ protocols. The extracted RNA was suspended in 50 *μ*l ultra-high quality diethyl pyrocarbonate-treated water.

### PCR for detection of HPV DNA

The presence and integrity of the DNA in all samples was verified by PCR β-globin gene amplification using the primers PC04 and GH20 (20). Water (negative control) and DNA from HPV-16-positive CaSki cells (positive control) were included in each amplification series. The presence of HPV DNA was analyzed by PCR using the general consensus primer sets GP5^+^/GP6^+^ and MY09/11 (21,22). DNA samples that were negative for HPV using GP5^+^/GP6^+^ or MY09/11 PCR were re-amplified by (auto-) nested PCR using the GP5^+^/GP6^+^ primer pair as previously described (23). PCR products were purified and directly sequenced with an ABI PRISM 3130xl Genetic Analyzer (Applied Biosystems, Foster City, CA). Obtained sequences were aligned and compared with those of known HPV types in the GenBank database using the BLAST program.

### Detection of HPV E6/E7 mRNA by reverse transcription PCR

Before cDNA synthesis, any residual DNA was removed by incubation with 1 U DNase I (Ambion) at room temperature for 25 min. cDNA was then synthesized from DNA-free total RNA using the RETROscript^®^ kit (Ambion) according to the manufacturer’s instructions. To examine the presence of contaminating DNA in RNA samples, all the assays were performed both with and without reverse transcriptase.

To detect high-risk *E6/E7* mRNA transcripts, PCR was performed with the cDNA from HPV DNA-positive samples using the Takara PCR Human Papillomavirus Typing Set (Takara, Bio Inc., Otsu, Shiga, Japan), which can identify high-risk HPV types 16, 18, 31, 33, 35, 52 and 58. To verify the HPV-16 *E6/E7* mRNA transcripts, the HPV-16 DNA-positive samples were also examined using a half-nested PCR approach with cDNA as previously described by Wiest *et al* (24). Positive PCR products were purified and directly sequenced using an ABI PRISM 3130xl Genetic Analyzer (Applied Biosystems).

### Immunohistochemistry for p16^INK4a^ and scoring of results

Serial 4-*μ*m-thick sections from FFPE tumor samples were deparaffinized in a graded alcohol series. Epitope retrieval was performed by heating at 95–99° C for 10 min in Tris/EDTA buffer (pH 9.0). Endogenous peroxidase activity was quenched by incubating the sections in 3% hydrogen peroxide plus 15 mM sodium azide for 5 min. The sections were subsequently incubated overnight at 4°C with primary monoclonal mouse anti-p16^INK4a^ antibody (MTM Laboratories AG, Heidelberg, Germany). After extensive washing in phosphate-buffered saline, the slides were incubated for 30 min at room temperature with a horseradish peroxidase-conjugated goat anti-mouse secondary antibody (MTM Laboratories). Immunolabeling was visualized by incubation in 3-3′-diaminobenzidine for 10 min. Stained slides were counterstained with hematoxylin.

Cases were considered p16^INK4a^-positive when intense nuclear and/or cytoplasmic reactivity was present. The scoring criteria for p16^INK4a^ immunoreactivity (p16^INK4a^ expression) were defined for this study based on previous scoring methods (14,15): 0 (no staining), 1 (1–10% of tumor cells positive), 2 (11–40% positive), 3 (40–70% positive) and 4 (>70% positive). The term ‘p16^INK4a^ Overexpression’ is defined as a score of 3 or 4.

### Survival analysis

Descriptive statistics were used to characterize patient baseline characteristics. The Mann-Whitney U-test or Kruskal-Wallis test was used for continuous variables, and Pearson’s χ^2^ test or Fisher’s exact test was used for categorical variables. The inter-rater agreements between HPV-DNA presence and p16^INK4a^ expression and between HPV mRNA expression and p16^INK4a^ expression were measured by calculating Cohen’s κ coefficient. A κ-value <0.20 was considered slight agreement, 0.21–0.40 as fair, 0.41–0.60 as moderate, 0.61–0.80 as good and 0.81–0.99 as excellent agreement (17,25–27).

Locoregional control was defined as complete and persistent disappearance of disease at the primary tumor (T site) and regional lymph nodes (N site) after treatment. Recurrence-free survival was defined as the time from the end of treatment to cancer recurrence or last follow-up. Disease-specific survival was defined as the time from the end of treatment to subsidence of disease or last follow-up. Survival curves were evaluated by the Kaplan-Meier method, and survival distributions were compared using the log-rank test. Multivariate Cox proportional hazard analysis was used to identify prognostic parameters and treatments associated with risk of recurrence and disease-specific death. P-values <0.05 were considered significant. All statistical analyses were performed using the SPSS statistical package (SPSS for Windows version 12.0; SPSS, Inc., Chicago, IL).

## Results

### Characteristics of eligible patients and follow-up

Primary tumor location was the nasopharynx in 10 patients (6.7%), oropharynx in 53 (35.3%), hypopharynx in 39 (26.0%), larynx in 24 (16.0%) and oral cavity in 24 (16.0%). The follow-up period ranged from 6 to 77 months, with a median of 38 months for patients whose data were censored. Sixty-four patients were treated with CCRT, 45 with surgery and postoperative RT or CRT (radiation dosage, 50–54 Gy), 22 with surgery alone and 19 with RT alone. Demographic and clinical characteristics are summarized in Table I.

### HPV DNA status and HPV E6/E7 mRNA expression

HPV DNA was detected by PCR in 47 of 150 (31.3%) primary untreated HNSCC specimens, including 30.0% (3/10) of nasopharyngeal, 47.2% (25/53) of oropharyngeal (18/30 of palatine tonsil), 17.9% (7/39) of hypopharyngeal, 16.7% (4/24) of laryngeal and 33.3% (8/24) of oral cavity cases. Among HPV-positive HNSCC samples, 39 (83.0 %) were infected with HPV-16 and the others were infected with other high-risk types (4 with HPV-33, 1 with HPV-35, 2 with HPV-58 and 1 with HPV-56).

As two of these HPV-positive samples were insufficient for RNA assay, *E6/E7* mRNA expression by HPV-16, HPV-33, HPV-35, HPV-58 and HPV-56 was examined by reverse transcription PCR in 45 samples. The *E6* and *E7* mRNA transcripts were detected in 21 of 45 (46.7%) specimens, the majority from OPSCC cases (18/25 HPV-positive cases), as shown in Table II.

Between the HPV DNA-positive and -negative groups, there were significant differences in the distribution of histological differentiation and tumor location; for example, the HPV DNA-positive group showed poor differentiation in histology and has a higher occurrence of oropharyngeal carcinoma compared with HPV DNA-negative group. The p16^INK4a^ overexpression group showed similar clinical characteristics (Table I).

### p16^INK4a^ expression and correlation with HPV status

**Figure 1.**
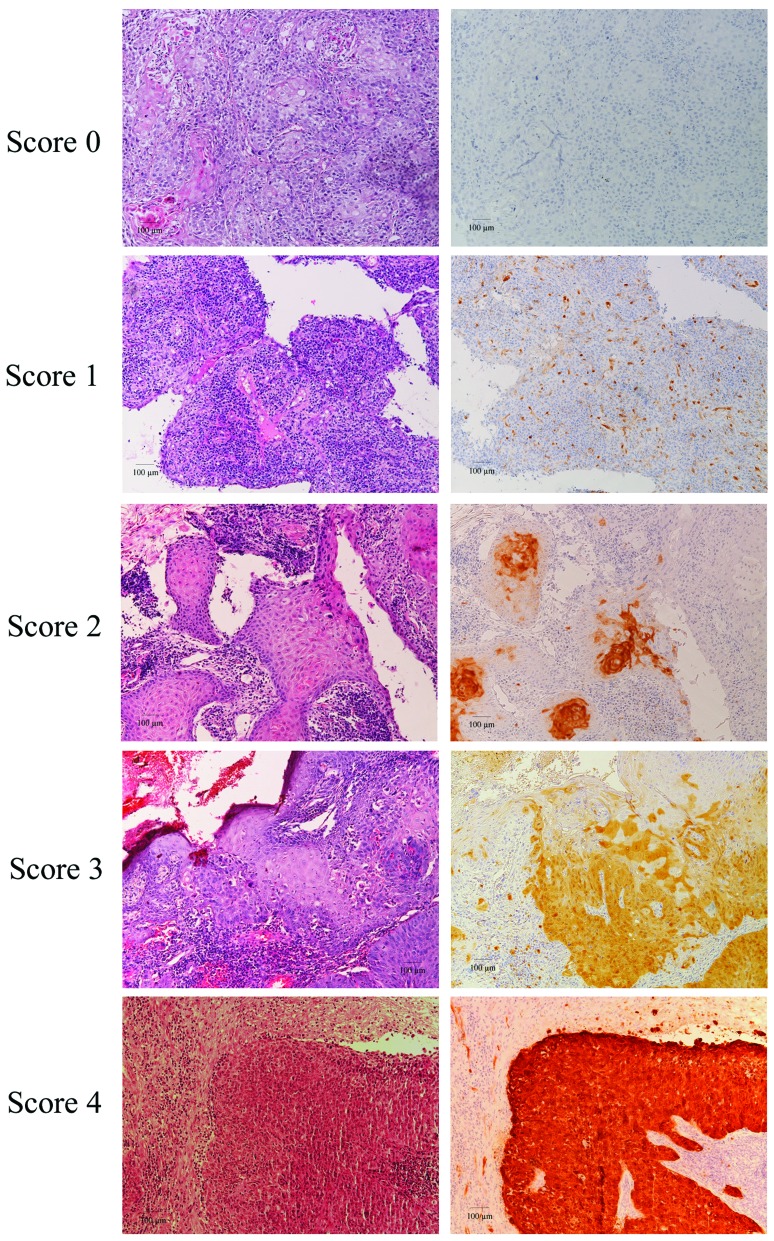
Immunohistochemical evaluation of p16^INK4a^ expression and scoring in HNSCC. p16^INK4a^ immunoreactivity was scored as 0 (no staining), 1 (1–10% of the tumor cells positive), 2 (11–40% of the tumor cells positive), 3 (40–70% of the tumor cells positive) or 4 (>70 of the tumor cells positive). Each micrograph shows the typical p16^INK4a^ immunoreactivity pattern corresponding to each score (A–F, ×100; bar, 100 *μ*m). Sections were also stained with hematoxylin and eosin (H&E).

In this study, the p16^INK4a^ expression scoring system (0–4) was based on the percentage of p16^INK4a^-positive cells (Fig. 1). Expression of p16^INK4a^ was observed in 40 of 150 (26.7%) HNSCC samples (Table III). The highest frequency of p16^INK4a^ expression was found in OPSCC samples, with 22 of 53 (41.5%) samples demonstrating a p16^INK4a^ staining score ≥1. The sensitivity of p16^INK4a^ staining for detection of HPV DNA in HNSCC was just 61.7%, as only 29 of 47 HPV-positive cases also had detectable p16^INK4a^ staining. The specificity was 89.3% (92/103 HPV-negative samples had a p16^INK4a^ staining score of 0) for all HNSCC cases (Table III). Although the correlation between p16^INK4a^ expression and HPV DNA and the correlation between p16^INK4a^ expression and *E6/E7* mRNA expression in all HNSCC cases proved to be significant (both P<0.001), the inter-rater agreements were relatively low (κ=0.53 and 0.54, respectively).

In contrast to general p16^INK4a^ expression, p16^INK4a^ over-expression demonstrated high specificity for detection of HPV DNA in HNSCC (98/103 HPV-DNA negative cases did not overexpress p16^INK4a^) and OPSCC (28/28 cases). The sensitivity of p16^INK4a^ overexpression was only 53.2% (25/47 cases) for detection of HPV DNA in HNSCC, but was considerably better for detection of HPV DNA in OPSCC at 80% (20/25 cases) (Table III). However, p16^INK4a^ overexpression indicated the presence of HPV *E6/E7* mRNA expression with high sensitivity at 90.5% (19/21) and high specificity at 91.3% (116/127) in HNSCC, and was even more accurate for OPSCC, with sensitivity at 94.4% (17/18) and specificity at 91.4% (32/35) (Table IV). Moreover, the inter-rater agreement of p16^INK4a^ overexpression for HPV *E6/E7* mRNA expression status was good for HNSCC (κ=0.69) and excellent for OPSCC (κ=0.84).

Based on a combination of HPV DNA status and p16^INK4a^ overexpression, subjects with OPSCC were divided into two groups: a double positive group of patients who were HPV DNA-positive with p16^INK4a^ overexpression and a single positive-negative or double negative group of patients who were either HPV DNA-positive without p16^INK4a^ overexpression, HPV DNA-negative with p16^INK4a^ overexpression, or HPV-DNAnegative without p16^INK4a^ overexpression. The combination of HPV DNA status and p16^INK4a^ overexpression had both high sensitivity (94.4%) and specificity (100%) for detecting HPV *E6/E7* mRNA expression in OPSCC (Table V).

### Prognostic value of HPV status and p16^INK4a^ overexpression in OPSCC

Within the observation period, 7 of the 53 patients (13.2%) developed recurrent disease, including 2 of 10 (20.0%) patients with stage I/II OPSCC and 5 of 43 (11.6%) patients with advanced stage OPSCC. However, no patient died with disease during the follow-up period. Since the disease specific rate and overall survival rate in OPSCC were quite fair in the present study, the prognostic analysis of OPSCC focused on recurrence-free survival. Since there were few recurrence-free survival events in OPSCC cases, multivariate analysis of recurrence-free survival was carried out instead for HNSCC cases overall.

**Figure 2.**
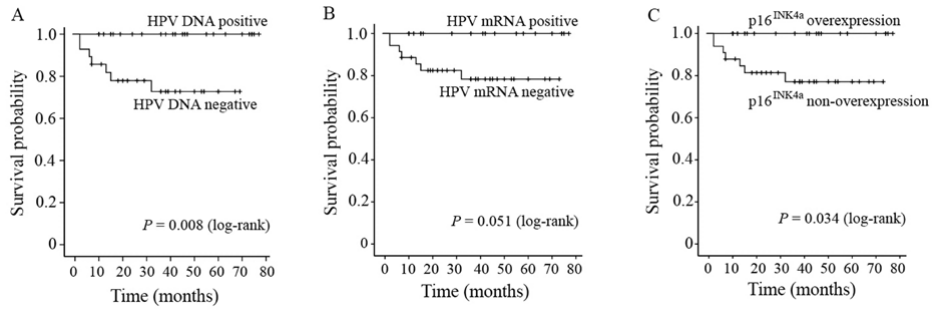
Kaplan-Meier curves of recurrence-free survival in OPSCC patients according to (A) HPV DNA, (B) HPV mRNA and (C) p16^INK4a^ overexpression. Recurrence-free survival was significantly better in HPV DNA-positive OPSCC patients than HPV DNA-negative OPSCC patients and in OPSCC patients with p16^INK4a^ overexpression than in OPSCC patients without p16^INK4a^ overexpression.

i) Prognostic value of HPV DNA status, *E6/E7* mRNA expression and p16^INK4a^ overexpression in OPSCC. HPV DNA-positive patients with OPSCC had better recurrence-free survival than HPV DNA-negative patients with OPSCC (P=0.008) (Fig. 2). The recurrence-free survival after 3 years was 72.7% for patients with HPV DNA-negative OPSCC and 100% for patients with HPV DNA-positive OPSCC. On the contrary, there were no significant differences in recurrence-free survival and disease-specific survival between HPV DNA-positive and -negative patients with non-OPSCC (P=0.139 and 0.144, respectively; Kaplan-Meier curves not shown).

HPV mRNA-positive OPSCC patients exhibited a trend toward improved recurrence-free survival compared with HPV mRNA-negative patients with OPSCC (P=0.051) (Fig. 2). The recurrence-free survival after 3 years was 78.3% for patients with HPV mRNA-negative OPSCC and 100% for patients with HPV mRNA-positive OPSCC.

**Figure 3.**
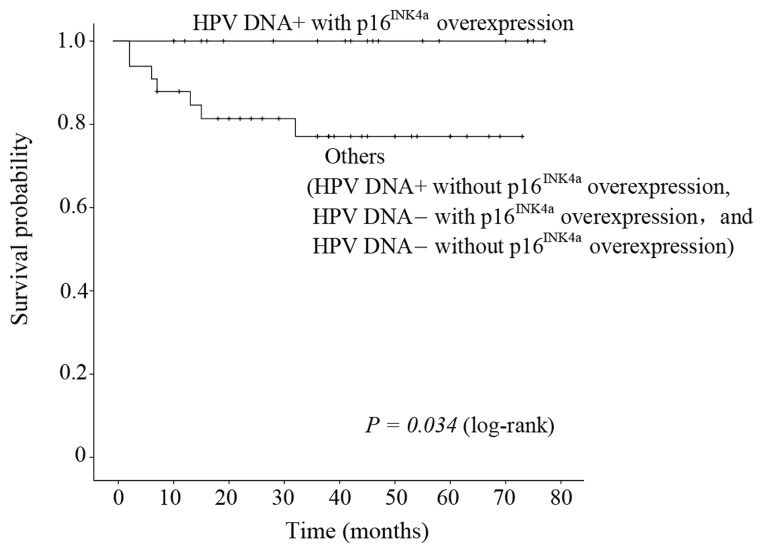
Kaplan-Meier curves of recurrence-free survival in OPSCC patients according to the combination of HPV DNA status and p16^INK4a^ overexpression. OPSCC patients with both HPV DNA-positive status and p16^INK4a^ overexpression showed significantly better recurrence-free survival compared to other patients (HPV DNA-positive without p16^INK4a^ overexpression, HPV DNA-negative with p16^INK4a^ overexpression and HPV DNA-negative without p16^INK4a^ overexpression (P=0.034).

OPSCC patients with p16^INK4a^ overexpression showed significantly improved recurrence-free survival compared with OPSCC patients without p16^INK4a^ overexpression (P=0.034) (Fig. 2). The recurrence-free survival after 3 years was 100 and 77.1%, respectively.

ii) Comprehensive evaluation of survival according to HPV DNA status and p16^INK4a^ overexpression in OPSCC. Patients in the double positive group had better recurrence-free survival than those in a single negative groups or the double negative group (P=0.034) (Fig. 3), with recurrence-free survival after 3 years of 100% and 77.1%, respectively.

iii) Multivariate analysis using Cox proportional-hazard model in HNSCC. To assess the independent predictive value of all these factors for recurrence-free survival in HNSCC, multivariate analysis using Cox proportional-hazards models was performed. Both HPV DNA presence and p16^INK4a^ over-expression was modeled as one factor. Since no patients with positive HPV *E6/E7* mRNA expression had any recurrent lesion, the influence of HPV mRNA expression on recurrence-free survival could not be evaluated.

In univariate analysis, the HNSCC patients with HPV DNA-positive status and p16^INK4a^ overexpression demonstrated significantly higher recurrence-free survival (P=0.015; hazard ratio (HR) = 8.61; 95% confidence interval (CI) = 1.12–66.18) compared with other HNSCC patients (Table VI). The HNSCC patients categorized in T stage 4 and those with oral cavity SCC had significantly lower recurrence-free survival (T4, P=0.003, HR=2.47, 95% CI=1.6–5.76; oral cavity SCC, P=0.031, HR=3.94, 95% CI=1.25–12.41) compared with patients categorized in T stages 1–3 and with OPSCC, respectively.

The final model of multivariate analysis using a Cox proportional hazards model for identification of fair recurrence-free survival of HNSCC showed that T1–3 stage (P=0.008; adjusted HR=2.61; 95% CI=1.29–5.29) and the combination of HPV DNA-positive status and p16^INK4a^ overexpression (P=0.043; adjusted HR=7.81; 95% CI=1.07–57.19) predicted significantly better recurrence-free survival (Table VI).

## Discussion

Over the past two decades, HR-HPV has been firmly established as a common etiologic factor in OPSCC and is now widely used as a prognostic marker for OPSCC. Although there are a number of studies on the epidemiologic role and prognostic value of HPV in OPSCC, some did not distinguish between patients receiving curative treatment from those receiving palliative care (28–31). Moreover, there are few studies on the prognostic value of HPV infection in non-oropharyngeal SCC. In this study, we analyzed the prognostic value of HPV infection in a retrospectively selected cohort of HNSCC patients receiving curative treatment. While the oropharynx was the site of highest prevalence (47.2%), this cohort also included patients with nasopharyngeal, hypopharyngeal, laryngeal and oral cavity tumors. The relatively high prevalence of HPV in cases of nasopharynx and oral cavity SCC suggests that HPV may play an important role in these non-oropharynx HNSCCs as well as in OPSCC.

A significant correlation was found between the presence of HPV DNA and improved recurrence-free survival in OPSCC, with HPV DNA-negative patients demonstrating an apparent greater risk of recurrence compared with HPV DNA-positive patients. OPSCC patients with HPV mRNA expression (active infection) also displayed improved recurrence-free survival compared with OPSCC patients without HPV mRNA expression (P=0.051), in line with previous studies (6,9,32–35). Although HPV DNA-positive patients with nonoropharyngeal SCCs also showed better recurrence-free and disease-free survival, the prognostic value did not reach statistical significance. Since OPSCC patients accounted for 34% of subjects in our series, the fair prognostic significance of HPV status for non-OPSCC HNSCC patients may be influenced by the generally excellent prognosis of patients with OPSCC. Indeed, Isayeva *et al* systematically reviewed the published data regarding the prognostic significance of HPV in SCCs of the oral cavity, larynx, sinonasal tract and nasopharynx and found no association between HPV status and treatment outcome (36). Further studies are needed to clarify the influence of HPV infection on prognosis in non-OPSCC cases.

Several studies have suggested that p16^INK4a^ expression can be used as a surrogate marker for HPV infection in OPSCC (14,37). Klaes *et al* defined p16^INK4a^ staining as negative, sporadic, focal or diffuse and found a significant correlation between the presence of HR-HPV and strong diffuse p16^INK4a^ expression (14). However, there are also several contradictory reports on the value of p16^INK4a^ as a biomarker. Smith *et al* found no concordance between p16^INK4a^ expression and HPV detection in 20% of head and neck cancers (38), possibly due to transcriptionally inactive infection or an alternate pathway of p16^INK4a^ activation (39). Using the same histological criteria as Klaes *et al* (14), Hoffmann *et al* reported that 81.2% of HPV DNA-positive HNSCC patients were also p16^INK4a^ positive, compared with only 48.2% of HPV DNA-negative cases, including 3 cases with strong and diffuse p16^INK4a^ staining. Moreover, no p16^INK4a^ expression could be detected in 3 of 14 HPV DNA^+^/RNA^+^ HNSCC lesions in their series. They concluded that p16^INK4a^ expression status alone is inadequate for identifying biological active or inactive HPV infections in HNSCC (40).

In the present study, the sensitivity of general p16^INK4a^ expression for detecting HPV DNA in HNSCC was also low, and there was generally low rate of agreement between p16^INK4a^-positive status and HR-HPV *E6/E7* mRNA expression in both HNSCC (κ=0.56) and OPSCC (κ=0.57). These results indicate that p16^INK4a^ expression alone is not suitable for identifying HPV-related tumors. However, p16^INK4a^ over-expression (p16^INK4a^ expression score ≥3) was a sensitive and specific marker for detecting HR-HPV mRNA expression in both HNSCC and OPSCC. This result also underscores the potential of our scoring system for evaluating p16^INK4a^ expression and determining prognosis.

Recent studies have demonstrated a significant correlation between p16^INK4a^ expression as a surrogate marker of HPV infection and fair prognosis in OPSCC. Lassen *et al* reported that p16^INK4a^-positive HNSCC showed a better response to conventional radiotherapy than p16^INK4a^-negative HNSCC, and ascribed this survival benefit to a better locoregional control rate (13). In a study by Fischer *et al* (18), p16^INK4a^-negative OPSCC patients demonstrated a more than 2-fold greater risk of death compared with p16^INK4a^-positive patients. Although locoregional OPSCC relapse was independent of p16^INK4a^expression, multivariate survival analysis indicated that p16^INK4a^ expression was an independent prognostic indicator for OPSCC, but not HNSCC, after adjustment for clinical T classification, clinical N classification, and treatment modality (18). In the present study, the combined evaluation of HPV DNA status and p16^INK4a^ overexpression could predict HPV *E6/E7* mRNA expression in OPSCC with both high specificity (100%) and sensitivity (94.4%). Furthermore, the combined evaluation of HPV DNA status and p16^INK4a^ overexpression was an independent prognostic indicator for HNSCC in multivariate analysis. Given the time and expense of *E6/E7* mRNA analysis, this combination may be particularly useful for larger scale clinical studies.

The combined evaluation of HPV DNA status and p16^INK4a^ overexpression was strongly correlated with HPV mRNA expression in OPSCC. Since the combination of HPV DNA-positive status and p16^INK4a^ overexpression showed a close relation with fair recurrence-free survival in HNSCC in multivariate analysis, the combination can serve as an accurate surrogate marker for biologically active HPV infection. This combined evaluation appears to be a useful and reliable method for detecting HPV-related HNSCC and determining its prognosis.

## Figures and Tables

**Table I.** Demographic and clinical characteristics.

Characteristic	Total no.	HPV^+^	HPV^−^	P-value	p16^INK4a^ overexpression		P-value
	
		n=47	n=103		(+) n=30	(-) n=120	
Gender, n (%)^*^							
Male	127	38 (29.9)	89 (70.1)	0.381	24 (18.9)	103 (81.1)	0.408
Female	23	9 (39.1)	14 (60.9)		6 (26.1)	17 (73.9)	
Age (years)							
Mean	64.1	62.6	64.8	0.286	61.8	64.7	0.216
Range	28–89	39–89	28–83		39–89	28–86	
≤50, n (%)	20	9 (45.9)	11 (55.0)	0.157	7 (35.0)	13 (65.0)	0.128
>50, n (%)	130	38 (29.2)	92 (70.8)		23 (17.7)	107 (82.3)	
Smoking, n (%)^a^				0.066			0.097
Never	30	14 (46.7)	16 (53.3)		10 (33.3)	20 (66.7)	
≤400	21	8 (38.1)	13 (61.9)		5 (23.8)	16 (76.2)	
>400	99	25 (25.3)	74 (74.7)		15 (15.2)	84 (84.8)	
Alcohol use, n (%)^b^				0.592			0.138
Never	25	10 (40.0)	15 (60.0)		6 (24.0)	19 (76.0)	
≤50	51	15 (29.4)	36 (70.6)		14 (27.5)	37 (72.5)	
>50	74	22 (29.7)	52 (70.3)		10 (13.5)	64 (86.5)	
T classification, n (%)				0.089			0.052
T1	18	2 (11.1)	16 (88.9)		1 (5.6)	17 (94.4)	
T2	58	22 (37.9)	36 (62.1)		17 (29.3)	41 (70.7)	
T3	41	10 (24.4)	31 (75.6)		5 (12.2)	36 (87.8)	
T4	33	13 (39.4)	20 (60.6)		7 (21.2)	26 (78.8)	
Node status, n (%)							
N0 or N1	83	24 (28.9)	59 (71.1)	0.477	17 (20.5)	66 (79.5)	0.870
N2 or N3	67	23 (34.3)	44 (65.7)		13 (19.4)	54 (80.5)	
TNM stage, n (%)							
Early (I and II)	42	9 (21.4)	33 (78.6)	0.103	8 (19.0)	34 (81.0)	0.856
Advanced (III and IV)	108	38 (35.2)	70 (64.8)		22 (20.4)	86 (79.6)	
Differentiation, n (%)				< 0.001			0.030
Well	68	13 (19.1)	55 (80.9)		8 (42.1)	11 (57.9)	
Moderate	63	21 (33.3)	42 (66.7)		12 (19.0)	51 (81.0)	
Poor	19	13 (68.4)	6 (31.6)		10 (14.7)	58 (85.3)	
Tumor location, n (%)				0.017			0.002
Hypopharynx	39	7 17.9)	32 (82.1)		3 (7.7)	36 (92.3)	
Oropharynx	53	25 (47.2)	28 (52.8)		20 (37.7)	33 (62.3)	
Oral cavity	24	8 (33.3)	16 (66.7)		2 (8.3)	22 (91.7)	
Larynx	24	4 (16.7)	20 (83.3)		3 (12.5)	21 (87.5)	
Nasopharynx	10	3 (30.0)	7 (70.0)		2 (20.0)	8 (80.0)	

a Brinkman index: daily cigarettes x years.

b Light drinker ≤50 g alcohol/day; Heavy drinker >50 g alcohol/day. HPV, human papillomavirus.

**Table II.** mRNA expression in HPV-DNA-positive HNSCC samples from various sites.

HPV	Site					Total (%)^a^
	NP (%)	OP (%)	HP (%)^a^	LC (%)^a^	OC (%)	
DNA^+^/mRNA^+^ (%)	1 (33.3)	18 (72.0)	0 (0)	1 (33.3)	1 (12.5)	21 (46.7)
DNA^+^/mRNA^−^ (%)	2 (66.7)	7 (28.0)	6 (100)	2 (66.7)	7 (87.5)	24 (53.3)

HPV, human papillomavirus; NP, nasopharynx; OP, oropharynx; HP, hypopharynx; LC, larynx; OC, oral cavity.

a Two samples (1 each from HP and LC) were insufficient for RNA assay.

**Table III.** Scoring of p16^INK4a^ overexpression and its association with HPV DNA in HNSCC and OPSCC.

	Scoring	p16^INK4a^ expression					Total
		p16^INK4a^ no or lower expression (<3)			p16^INK4a^ overexpression (≥3)		
		0	1	2	3	4	
HNSCC	HPV DNA^+^	18	3	1	1	24	47
	HPV DNA^−^	92	3	3	1	4	103
OPSCC	HPV DNA^+^	4	1	0	1	19	25
	HPV DNA^−^	27	0	1	0	0	28

HPV, human papillomavirus; HNSCC, head and neck squamous cell carcinoma; OPSCC, oropharyngeal squamous cell carcinoma.

**Table IV.** Correlation between p16^INK4a^ overexpression and HPV mRNA in HNSCC and OPSCC.

p16^INK4a^ overexpression	HPV mRNA		Sensitivity (%)	Specificity (%)	PPV (%)	NPV (%)
	+	-				
HNSCC						
+ (≥3)	19	11	90.5	91.3	63.3	98.3
− (<3)	2	116				
OPSCC						
+ (≥3)	17	3	94.4	91.4	85.0	97.0
− (<3)	1	32				

HPV, human papillomavirus; HNSCC, head and neck squamous cell carcinoma; OPSCC, oropharyngeal squamous cell carcinoma; PPV, positive predictive value; NPV, negative predictive value.

**Table V.** Relationship between HPV *E6/E7* mRNA expression and HPV DNA/p16^INK4a^ expression status in OPSCC.

	HPV mRNA expression		P-value
HPV DNA/p16^INK4a^ expression status	+	−	
HPV DNA^+^ with	17	0	<0.001
p16 overexpression			
Others	1	33	
HPV DNA^+^ without p16 overexpression	1	5	
HPV DNA^−^ with p16 overexpression	0	0	
HPV DNA^−^ without p16 overexpression	0	28	

**Table VI.** Univariate and multivariate analysis demonstrating the prognostic impact of HPV DNA and p16^INK4a^ overexpression on recurrence-free survival in HNSCC.

Variable	P-value	Univariate analysis (n=150) HR (95% CI)	P-value	Multivariate analysis (n=150) HR (95% CI)
HPV DNA/p16^INK4a^ expression (i.e., HPV^+^ with p16^INK4a^ vs. others)	0.015	8.61 (1.12–66.18)	0.043	7.81 (1.07–57.19)
Age (≤50 vs. >50 years)	0.399	0.64 (0.23–1.82)		
Gender (male vs. female)	0.334	0.62 (0.23–1.65)		
T stage (T4 vs. T1–T3)	0.033	2.47 (1.06–5.76)	0.008	2.61 (1.29–5.29)
Nodal stage (N2 or N3 vs. N0 or N1)	0.942	0.97 (0.45–2.10)		
Smoking (yes vs. never)	0.559	0.76 (0.30–1.91)		
Alcohol consumption (yes vs. never)	0.222	0.56 (0.22–1.44)		
Differentiation				
Well				
Moderate	0.312	0.65 (0.29–1.50)		
Poor	0.770	0.74 (0.22–2.53)		
Tumor location				
Oropharynx		Reference		Reference
Nasopharynx	0.063	4.38 (0.98–19.52)	0.317	1.92 (0.54–5.89)
Hypopharynx	0.217	1.97 (0.66–5.86)	0.865	1.09 (0.40–3.02)
Larynx	0.500	1.73 (0.49–6.13)	0.870	1.10 (0.35–3.50)
Oral cavity	0.031	3.94 (1.25–12.41)	0.107	2.27 (0.84–6.17)

HPV, human papillomavirus; HNSCC, head and neck squamous cell carcinoma; HR, hazard ratio; CI, confidence interval.

## Abbreviations:

CCRT: concurrent chemoradiotherapy;
CI: confidence interval;
CRT: chemoradiotherapy;
FFPE: formalin-fixed, paraffin-embedded;
HNSCC: head and neck squamous cell carcinoma;
HPV: human papillomavirus;
HR: hazard ratio;
HR-HPVs: high-risk HPV variants;
OPSCC: oropharyngeal squamous cell carcinoma;
PCR: polymerase chain reaction;
RT: radiation therapy
